# Physical Activity Drops During Summer Holidays for 6- to 9-Year-Old Children

**DOI:** 10.3389/fpubh.2020.631141

**Published:** 2021-01-18

**Authors:** Tadeja Volmut, Rado Pišot, Jurij Planinšec, Boštjan Šimunič

**Affiliations:** ^1^Faculty of Education, University of Primorska, Koper, Slovenia; ^2^Science and Research Centre Koper, Institute for Kinesiology Research, Koper, Slovenia; ^3^Faculty of Education, University of Maribor, Maribor, Slovenia

**Keywords:** MVPA, physical inactivity, sedentary behavior, vacations, accelerometer

## Abstract

Regular physical activity (PA) reduces the health risk of childhood obesity and associated chronic diseases as well as mental health problems. Since PA declines as children age as well with future generations it is of highest importance to intervene in school and out-of-school settings. Out-of-school periods affect children's PA as it is mainly left to the interest and motivation of their parents. We compared accelerometer-based PA patterns in 93 6- to 9-year old children assessed four times: before (May/June), during (August), and after (September) summer holidays and at a 1-year follow up (May/June). Before summer holidays children were assessed also for anthropometry and motor tests. During summer holidays overall PA decreased by 18% (*p* < 0.001), physical inactivity increased by 5.5% (*p* < 0.001), moderate PA decreased by 53% (*p* < 0.001) and moderate to vigorous PA decreased by 45% (*p* < 0.001) when compared to before summer holidays. Furthermore, overall PA remained diminished also after summer holidays by 8.8% (*p* = 0.001) but recovered to baseline values at 1-year follow up. About 30% of overall PA and moderate to vigorous PA decrease during summer holidays could be explained by children's fitness level as a greater decrease was found in children with better results in standing long jump and 300-meter running time. Our finding detects an alarming summer holiday decrease in children PA that should not be neglected in future studies and intervention designs.

## Introduction

Regular participation in physical activity reduces the health risk of childhood obesity and associated chronic diseases as well as mental health problems (1, 2). Therefore, it is of the highest importance to establish optimal physical activity patterns in childhood that are associated with future health and well-being (3). Further, it is well-documented that physical activity declines as children age (4, 5) and with future generations (6).

There are many original (7–13) and review (14–26) articles reporting seasonal variations in children's physical activity. A vast majority (83%) of these confirm seasonal changes in physical activity, indicating that a single assessment will not adequately characterize children's physical activity habits (9, 11). Seasonal variations and the validity of results depend on several factors (11, 27, 28): region where children reside (especially in regard to cold regions), children's age (especially 8–12 years), physical activity assessment method (most valid results from accelerometer-based studies) and study design (most valid results from repeated measures studies). Of the vast majority of studies reporting seasonal variations from 2 to 5 assessments, there are only four studies that compared physical activity and inactivity during summer holidays vs. school time (15, 18, 24, 26), and only three were longitudinal studies (18, 22, 24).

When comparing changes in physical inactivity and activity from school time to summer holidays, the results are not consistent (18, 22, 24, 26). Although sedentary behavior or physical inactivity consistently increased during summer holidays in all four studies, from 1.7 to 8%, it is less clear for light physical activity (LPA), which decreased during summer holidays by 2–10% (18, 22, 24) or even increased by 9% (26). Similarly, decreases in moderate physical activity (MPA) in the range of 3–13% are reported in only two studies (18, 22) as well as a 16% decrease in moderate to vigorous physical activity (MVPA) (22). Those discrepancies could be due to the low number of participants (18, 24), wrist accelerometer wear (24); comparison to different baseline seasons—some with more than one assessments during the school term (26), others in spring (22, 24), some in summer (18), some in winter (26). Thus, such an important and worrying modulation of physical activity during summer holidays warrants further investigation.

Summer holidays changes in physical activity and sedentary behavior could be affected by many factors, including sex, age, body-mass index or fat mass, fitness level, climate, and/or region, the school education system, family socio-economic status, parents' availability, and motivation for physical activity, availability of summer sport programmes, etc. Tanaka et al. (22) reported lower increase in sedentary behavior for girls involved in summer sports and boys without bedroom television ownership. Similarly, MVPA decreased less in boys involved in summer holidays sport programmes than in those who did not. To support this, Brazendale et al. (24) reported almost double increase (97.5% being > 4 h/day) in screen time during summer holidays in comparison to school time. The magnitude of the summer holidays effect could be reflected also in the increase of body mass index percentile per age after only 2 months of summer holidays (18). However, it remains to be seen whether children's fitness plays a role in preserving physical activity and whether inactivity changes during summer holidays.

Only one study investigated summer holidays' effect with multiple assessments during the school time. As we know, there is a substantial lowering of the physical activity in children with their increasing age (4, 5). Volmut et al. (4) reported a 40% decline in overall physical activity in children after the age of 4 years until the age of 16 years. Therefore, a decline of 3.3%/year is expected in longitudinal studies. Researchers must be aware of this and investigate summer holidays changes with multiple assessments before and/or after summer holidays. Only one cross-sectional study has followed this (26) and compared average school time data with summer holidays assessment. However, there are no longitudinal studies with more than two assessments.

Therefore, the aim of this study is to compare longitudinal changes in physical activity and inactivity within four assessments (three during school time and one during summer holidays) and investigate whether changes are associated with children's fitness. We hypothesized that summer holidays will increase physical inactivity and decrease MVPA, whereas greater changes will be observed in less fit children. Second, we will investigate whether physical activity and inactivity changes are reversible after summer holidays.

## Materials and Methods

### Participants

Initially 100 children (aged from 6- to 9-years; 49% boys) participated in the study. After data validation, 93 participants reached valid initial assessment and were invited for the next assessments (Table 1). They were randomly and evenly recruited from nine Slovenian schools, from two major cities: Maribor (28%) and Ljubljana (36%); and three cities from the coastal region (Koper, Piran, and Izola) (36%). Schools, parents, and children were pre-informed of the protocol and written consent was obtained from parents prior to the data collection. The study was approved by the Slovenian National Medical Ethics Committee (approval number: 153/07/09). The study was performed in accordance with the ethical standards of the 1964 Declaration of Helsinki and later amendments.

**Table 1:** Participants of the study.

	**BSH**	**DSH**	**ASH**	**FU**
*N* (% boys)	93 (52)	46 (59)	81 (54)	56 (57)
**Anthropometry**				
Age/years	7.55 (1.14)			
Body height/m	1.28 (0.08)			
Body mass/kg	27.7 (5.84)			
Body mass index/kg/m^2^	27.7 (2.08)			
% increased health risk	10.3%			
Fat mass/%	20.0 (4.92)			
**Motor tests**				
Standing long jump/cm	119 (19.3)			
Coordination test/sec	7.85 (2.08)			
300-meter running/sec	90.4 (15.4)			

*BSH, before summer holidays; DSH, during summer holidays; ASH, after summer holidays; FU, 1-year follow up; Increased health risk was estimated from fat mass cut-off values (boys > 25%; girls > 30%) (29)*.

### Study Design

A longitudinal study design was used to assess physical activity in four time points in a 1-year period. Initial assessment took place during the school period in May/June (before summer holidays—BSH), the second assessment took place during summer holidays (DSH) in August, the third assessment took place during the school period in September (after summer holidays—ASH), and the fourth follow-up assessment took place again during school time in May/June (FU). At BSH children were assessed for anthropometrics and motor efficiency (standing long jump, 300-meter running test, and coordination test). As evident from Table 1, not all children attended all four measurements, whereas 46 (59% boys) were consistently assessed.

### Measurement Procedures

At BSH children were assessed for anthropometrics and motor development only at BSH, while physical activity was assessed at BSH and later at DSH, ASH, and FU.

*Anthropometrics* was assessed as body height and mass using standard tools with 0.5 cm and 0.1 kg accuracy, respectively. Further, a body mass index (BMI) was calculated. Fat mass was assessed with a bioimpedance analyzer (Bioscan 916s, Maltron, UK). Four electrodes were applied according to the manual, two to the instep of the right foot, one to the back of the right hand, and one to the right wrist. Thirty minutes before and during the assessment children lay supine on a bed. Assessment was performed in the morning hours, before breakfast and before any moderate or intense physical exercise.

*Motor efficiency* was assessed by the means of motor tests during physical education hours. Children were assessed after a standardized 15 min warm-up, consisting of 5 min of running, 5 min of stretching, and 5 min of specific drills for three motor tests. Standing long jump was performed barefoot on a special mat with marked distances. An experienced researcher with the help of a physical education teacher assessed the results. Each child performed two warm-up jumps and three test jumps, the longest was taken for further analysis. The coordination test consisted of walking backward through three vertical rings, with legs and hands touching the floor. The distance between the starting line and the first ring was 1 meter and the 80 cm rings were 1 meter apart. The time was measured from the starting signal until the child exited the last ring. Each child performed two warm-up trials and two test trials, the shortest time was taken for further analysis. The 300-meter running test was performed outside at the local stadium. Children were instructed to run 300 meters as fast as possible and the time was recorded for further analysis.

*Physical activity* was assessed by means of accelerometers (GT3x, Actigraph, USA) during 5 consecutive days (from Wednesday to Sunday). Accelerometers were pre-programmed for 1 min epochs and attached with elastic strap on the right hip. Parents and children received instructions for accelerometer wear: not to be used during sleep, bathing and swimming; to be worn from 8 a.m. until 8 p.m. After each assessment period the data were transferred to the computer and further analyzed in Matlab (R2009a, Mathworks, USA). Only data from 8 a.m. and 8 p.m. (12 h) were analyzed with a removal of all 20 min consecutive zeros. Inclusion criteria for data validation were: (i) at least 80% of wearing time (9.6 h) for a valid day, and (ii) at least 2 valid weekdays and 1 valid weekend day for a valid record (30). From a valid record an overall physical activity in counts per minute (cpm) was calculated. Further, time spent in each physical activity phenotype was calculated based on Puyau et al. (31) cut-off values established for 6–16-year-old children. Specifically, a cut off 800 cpm was used to distinguish physical inactivity (PI) and LPA, a cut-off of 3,200 cpm was used to distinguish between LPA and MPA, and a cut-off 8,200 cpm was used to distinguish between MPA and VPA.

### Statistics

All data were checked for normal distribution and normality was confirmed by visual inspection of histograms, Q-Q plots, kurtosis and skewness analysis (being lower than two standard errors of kurtosis and skewness), respectively, and the Shapiro-Wilk test confirmed normal distribution. The Leven test confirmed the equality of variances. Therefore, data are presented by means (with standard deviations) or [95% confidence intervals]. Using 3-way repeated analysis of variance we checked assessment time, age and sex differences. After dismissing age and sex differences we rearranged the physical activity data for mixed linear modeling where normality of residuals was confirmed by the same procedures as for the main variables. Subjects were classified as random factor, whereas assessment time point (BSH, DSH, ASH, FU), sex (boys and girls), age (6-, 7-, 8-, and 9-years) and city (coast, Ljubljana, and Maribor) were classified as fixed factors. In case of significant main effect, we applied a *post hoc* analysis with the Bonferroni correction of *p*-value to compare averages of two assessment points. Correlation analyses between changes in physical activity phenotypes during summer holidays and motor tests were performed using the Pearson correlation coefficient by applying the Bonferroni correction of *p*-value. All statistical decisions were made at *p* ≤ 0.05.

## Results

After applying the Bonferroni correction (α = 0.0025) we could not detect any sex or age differences in physical activity phenotypes at any time points. Therefore, we pooled the sample for further analysis (Table 2).

**Table 2:** Mean (with standard deviation) values for pooled and per gender participants assessed at baseline, before summer holidays.

	**Pooled**	**Boys**	**Girls**
*N*	93	48	45
Overall physical activity/cpm	750 (182)	772 (158)	726 (203)
Physical inactivity/min	504 (46.0)	493 (45.3)	517 (44.0)
Light physical activity/min	188 (39.0)	199 (38.8)	177 (36.1)
Moderate physical activity/min	25.1 (13.3)	25.1 (13.3)	23.8 (12.0)
Vigorous physical activity/min	2.0 (3.8)	1.36 (2.71)	2.7 (4.7)
Moderate and vigorous physical activity/min	27.1 (14.5)	27.8 (14.7)	26.4 (14.4)

After performing mixed linear modeling, we found assessment time contribution to the overall model in all physical activity phenotypes (*p* < 0.001). Sex contributed only in physical inactivity (*p* = 0.004) and LPA (*p* = 0.005), while age contributed in overall physical activity (*p* = 0.018), physical inactivity (*p* = 0.006), and LPA (*p* = 0.002). Geographical place of assessment (city) contributed only in VPA (*p* = 0.041).

**Figure 1:**
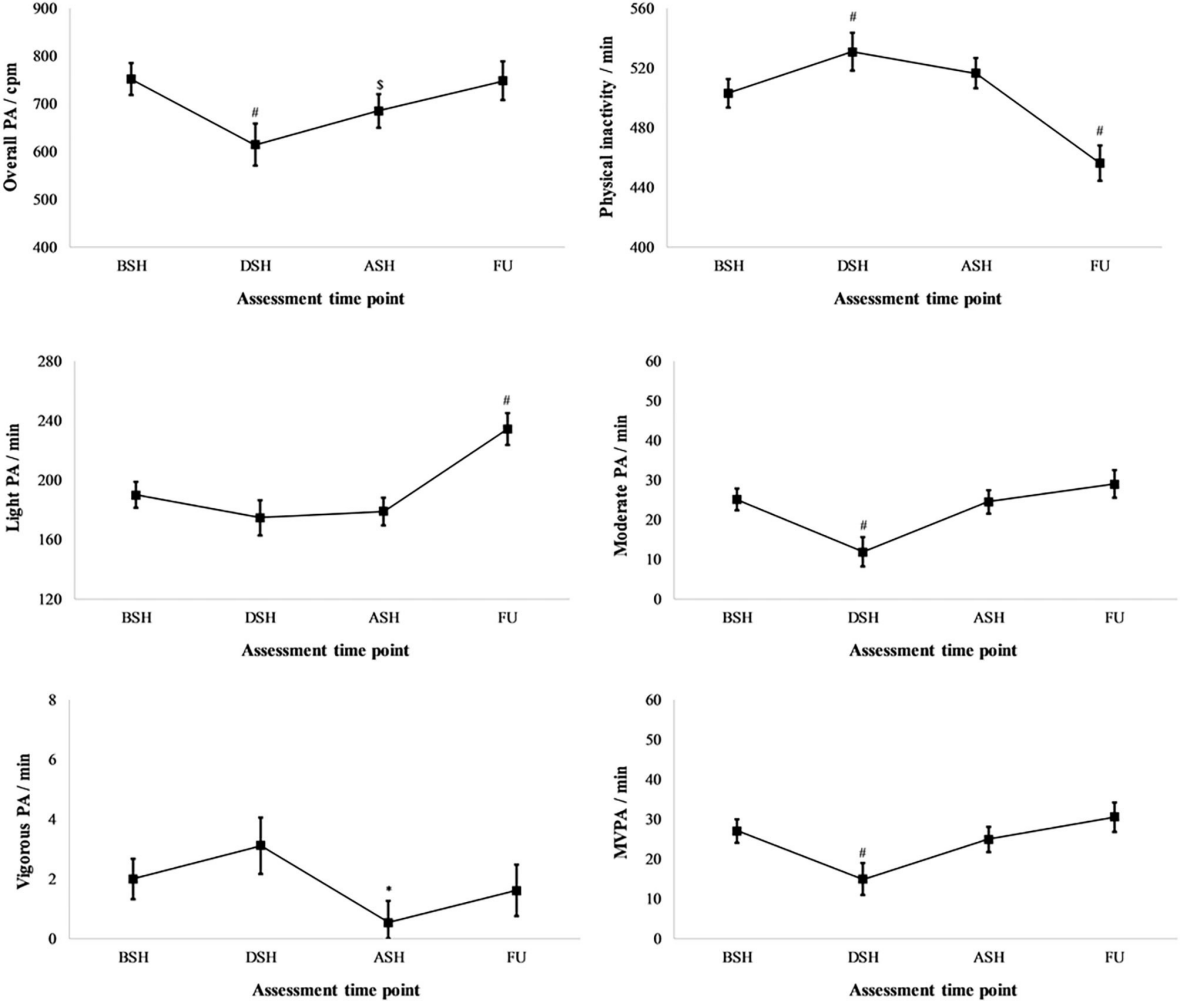
Changes in physical activity (PA) phenotypes during all four assessments: before (BSH), during (DSH), after (ASH) summer holidays and at 1-year follow up (FU). MVPA, moderate and vigorous physical activity. *p < 0.05; ^$^*p* < 0.01; ^#^*p* < 0.001.

Figure 1 presents changes in physical activity phenotypes when compared to baseline (BSH). An overall physical activity decreased by 18% in DSH (*p* < 0.001) remained decreased in ASH (8.8%; *p* = 0.001). PI increased by 5.5% (27.7 min) in DSH (*p* < 0.001), returned to baseline at ASH; however, in this FU it decreased by 9.3% (47 min) (*p* < 0.001). This decrease in PI at FU must be associated with a 23% (44 min) increase in LPA at the same time point (*p* < 0.001). MPA decreased by 53% (13 min) in DSH (*p* < 0.001) but not VPA, which decreased by 73% (1.5 min) in ASH (*p* = 0.011). Although large relative, but small absolute changes in VPA were found at ASH, this did not affect MVPA, which decreased by 45% (12 min) in DSH only (*p* < 0.001).

Due to high multicollinearity between motor tests (|r|>0.500), only bivariate correlations could be presented (Table 3). We have found that relative changes in overall physical activity between BSH and DSH were correlated to the standing long jump test (*r* = −0.413; *p* = 0.004) and the 300-meter running test time (*r* = 0.421; *p* = 0.004) but not to BMI or fat mass. Interestingly, relative changes in PI between BSH and DSH were not correlated to any motor test or anthropometric measure. However, relative changes in MVPA between BSH and DSH were also correlated to the 300-meter running test time (*r* = 0.544; *p* < 0.001).

**Table 3:** Pearson correlation (r) analysis of relative decreases during summer holidays (DSH) when compared to before summer holidays (BSH) with motor tests and anthropometric measures.

	**Overall physical activity decrease**	**Physical inactivity increase**	**MVPA decrease**
**Anthropometric measures**			
Fat mass	*r* = −0.080	*r* = −0.165	*r* = −0.094
Body mass index	*r* = 0.053	*r* = −0.084	*r* = 0.024
**Motor tests**			
Standing long jump	*r* = −0.413^$^	*r* = 0.185	*r* = −0.320
300-meter running	*r* = 0.421^$^	*r* = 0.048	*r* = 0.544^#^
Coordination	*r* = 0.104	*r* = 0.089	*r* = 0.323

$ p < 0.01;

# *p < 0.001*.

## Discussion

We compared longitudinal changes in physical activity and inactivity during a period of 12 months, including four assessments, three during school time (BSH, ASH, and FU), and one during summer holidays (DSH). When compared to BSH, we found that DSH overall physical activity decreased by 18%, together with 5.5% increased PI, 53% decreased MPA, and most importantly, with 45% decreased MVPA. We could conclude that physical activity phenotypes declined importantly in DSH. Even more, overall physical activity is not restored at ASH, being still lower by 8.8% than at BSH; however, it is restored at FU.

Although the summer months have been shown to be a time period when physical activity levels tend to be higher in children (28, 32), some well-designed longitudinal studies have showed that physical activity may actually be compromised during the summer holidays—as found also in our study. Although decreased MVPA during summer holidays is in line with the previous study by Tanaka et al. (22), we found a much greater decrease (45%, theirs being 16%). While increased PI was comparable in both studies (our study: 5.5% vs. 22: 4%) it seems that decreased MPA is a main contributor to the decrease in MVPA at DSH. However, in the study by McCue et al. (18), decreased MPA was not reflected in decreased MVPA. Tanaka et al. (22) reported results obtained from a similar sample at the same time-periods and interpreted the decline in MVPA at DSH due to higher maximum temperatures during the summer. Indeed Lewis et al. (33) reported that daily maximum temperature is significantly associated with MVPA and physical inactivity in Australia and Canada. MVPA and physical inactivity times appear to be optimal when the maximum temperature ranges between 20 and 25°C in both countries (33). This seems to hold for the study by Tanaka et al. (22), in which the average maximum daily temperature during the summer assessment was 31.6 (3.3) °C, with MVPA decreasing by 16% and PI increasing by 4%. Furthermore, in our study the average maximum daily temperature during the summer assessment was even higher, being 33.6 (1.3) °C, and the MVPA decrease was also the largest (45%); PI increased the most (5.5%), as well. During hot summer days, children tend to stay indoors to avoid heat and sun and that affects their physical activity (34, 35) and the outdoor physical activity in 6–9-year-old children is mainly left to the interest and motivation of their parents.

Previously published studies investigating the variability of children's physical activity in relation to the season are inconsistent. Most of the studies have shown seasonal differences in children's physical activity (9–11, 13, 14, 16, 17, 21, 22, 27, 28). After summarizing results, PI increases DSH from 1.7% (24) to 8% (18), while LPA decreases from 2% (23) to 10% (22), and MVPA increases from 8% (26) to 15.5% (22).

Other studies did not report change in MVPA during summer holidays. Although McCue et al. (18) found increased PI (16%), and decreased LPA (12%), and MPA (43%), there were no changes in VPA and MVPA. VPA remained unchanged in our study, too, although it was found to be very short in both studies (e.g., only 1–3 min). McCue et al. (18) suggested that the increased screen time might be an important contributor of increased sedentary behavior during summer holidays (36, 37); however, this was later dismissed by Brazendale et al. (24) when they found a 2-fold increased screen time without MVPA change and only 2.2% increased PI during summer holidays.

During summer holidays children have less-structured routines, and potentially less supervision throughout the day compared to their school schedules (37, 38). Indeed, all our participants assessed at DSH were involved in at least one organized out-of-school sport exercise in sport clubs. However, those activities were stopped at DSH, being continued at ASH, as goes for all school curricular and extracurricular physical activity or sport related activities.

Increased MVPA (24%), LPA (9%), and decreased PI (8%) at DSH were found by Nagy et al. (26). Although this seems to contradict our study and other studies (18, 22, 23), it should be noted that the study was cross-sectional and the reference values used in the study of Nagy et al. (26) were taken for the winter season. And it is well-known that physical activity is the lowest during winter (21, 28).

We should not neglect the fact that the overall physical activity remained down 8.8% at ASH compared to BSH. A similar finding was reported by Fu et al. (25), that reported 9% decreased daily steps immediately after 12 week summer holidays. The lower physical activity immediately after summer holidays may be reflected by the summer holiday period and additionally by the beginning of the new school year itself, as teachers may not have fully implemented physical activity breaks, physical education lessons may not have commenced, and plans to provide children with physical activity opportunities at recess have yet to be developed.

In recent years an accelerometer has been widely used for estimating physical activity, but we must point out that the accelerometer is not waterproof, to be used during water-based activities, which occurs most frequently during the summer holidays. This could add to the decline (or perception of decline) in physical activity during the summer, especially in our sample of children recruited also from coastal cities (Koper, Piran, and Izola). We have consistently used 1 min epochs and this might affect our results: overestimation of LPA, MPA, MVPA and underestimates PI and VPA in children (39). Furthermore, accelerometers were found to underestimate physical activity demand during cycling (40) and uphill hiking (41), both very popular physical activities in Slovenia. Perhaps this is one of the reasons why our participants have a higher decline in physical activity during summer periods.

We have found that declines during summer holidays, in comparison to BSH, were correlated to children's results in motor tests but not anthropometric measures. Generally, we found that better results in two motor tests (standing long jump and 300-meter running time) were correlated to a higher decrease in overall physical activity and MVPA but not to PI changes. This is the first study to evaluate motor tests as factors of physical activity decline during summer holidays. Although correlations were moderate, they explained the important 30% summer holidays decrease in MVPA variation.

Summer holidays, based on our results, are a time of large reduction in physical activity and an increase in physical inactivity in Slovenian children. This raises the question whether the same results would be achieved with older children and adolescents, as we know children and adolescents are undergoing an annual decrease in physical activity of up to 3.3% (4).

Our study was conducted prior to the COVID-19 epidemic. However, in the spring of 2020, we were hit by a global epidemic of the COVID-19, which brought lower physical activity, less time outdoors, higher sedentary behavior (including screen leisure time) in children and adolescents (42, 43). In addition, during the restrictive measures (closure of sport activities) due to the COVID-19 outbreak, up to 29% of children and adolescents showed no interest in continuing to engage in sport after the end of the first wave COVID-19 epidemic (44). It is obvious that such global effects need to be intervened on to assure health and motor efficiency of new-generation children.

We could conclude that physical activity decreases importantly during summer holidays and remains decreased after summer holidays. The greatest effect of summer holidays was found in decreased MVPA; however, increased physical inactivity should not be neglected. Although at the start of new school year the FU effects of summer holidays were restored, it should be noted, that overall physical activity remained 8.8% lower than before the summer holidays.

## Data Availability Statement

The raw data supporting the conclusions of this article will be made available by the authors, without undue reservation.

## Ethics Statement

The studies involving human participants were reviewed and approved by Slovenian National Medical Ethics Committee (approval number: 153/07/09). Written informed consent to participate in this study was provided by the participants' legal guardian/next of kin.

## Author Contributions

TV and BŠ: conception of the study, data assessment and analysis, and writing the manuscript. RP and JP: conception of the study and writing the manuscript. All authors contributed to the article and approved the submitted version.

## Conflict of Interest

The authors declare that the research was conducted in the absence of any commercial or financial relationships that could be construed as a potential conflict of interest.
